# Dog cloning with *in vivo* matured oocytes obtained using electric chemiluminescence immunoassay-predicted ovulation method

**DOI:** 10.1371/journal.pone.0173735

**Published:** 2017-03-13

**Authors:** Seunghoon Lee, Minghui Zhao, Jingu No, Yoonseok Nam, Gi-Sun Im, Tai-Young Hur

**Affiliations:** National Institute of Animal Science, RDA, Wanju, Republic of Korea; Peking University Third Hospital, CHINA

## Abstract

Radioactive immunoassay (RIA) is a traditional serum hormone assay method, but the application of the method in reproductive studies is limited by the associated radioactivity. The aim of present study was to evaluate the reliability of RIA and to compare its canine serum progesterone concentration determination accuracy to that of the electric chemiluminescence immunoassay (ECLI). *In vivo* matured oocytes were utilized for canine somatic cell nuclear transfer (SCNT), and serum progesterone levels were assessed to accurately determine ovulation and oocyte maturation. Canine serum progesterone concentrations during both proestrus and estrus were analyzed by RIA and ECLI to determine the ovulation day. Although both methods detected similar progesterone levels before ovulation, the mean progesterone concentration determined using ECLI was significantly higher than of RIA three days before ovulation. Following ovulation, oocytes were collected by surgery, and a lower percentage of mature oocytes were observed using ECLI (39%) as compared to RIA (67%) if 4-8ng/ml of progesterone were used for determination of ovulation. A high percentage of mature oocytes was observed using ECLI when 6–15 ng/mL of progesterone was used for ovulation determination. To determine whether ECLI could be used for canine cloning, six canines were selected as oocyte donors, and two puppies were obtained after SCNT and embryo transfer. In conclusion, compared to the traditional RIA method, the ECLI method is a safe and reliable method for canine cloning.

## Introduction

Several animals have been produced by nuclear transfer since the first successful cloning of an animal by somatic cell nuclear transfer (SCNT) was performed in 1996 [1], including cattle [2], sheep [3], pigs [4], mice [5], rats [6], rabbits [7], camels [8], and dogs [9]. The in vitro maturation (IVM) system of oocytes was developed as the major oocyte source for assisted reproduction, because of the low efficiency of SCNT and the limited quantity of in vivo matured oocytes. Although the IVM system was established in several species, the maturation rate in canine oocytes cultured in vitro remains low because of unique canine theriogenology. In order to produce more elite cloned canines, in vivo matured oocytes were employed for canine SCNT.

Unlike other mammalian oocytes, dog oocytes mature after ovulation in the oviduct [10]. After ovulation, the oocytes resume meiotic division and progress to the metaphase II stage within three days. Oocyte maturation is regulated by the maturation promoting factor (MPF, also called the mitosis-promoting factor), which is a complex of cyclin-dependent kinase 1 (CDK1) and cyclin B [11]. The MPF activity reaches its highest level at the MII stage, and the MPF activity level undergoes a time-dependent decrease after maturation [12]. Low MPF activity results in oocyte aging, which is presented as reduced developmental potential [13] and directly reduced embryonic development after nuclear transfer [14,15]. Therefore, an accurate method for evaluating the maturation of oocytes *in vivo* should be developed to obtain high quality oocytes for canine cloning.

For over 40 years, serum progesterone (P4) has been used to detect ovarian characteristics, estrous detection, and pregnancy in humans [16], cattle [17], mares [18], pigs [19], cats [20], and dogs [9]. The determination of the ovulation time is of major importance to reproductive management associated with artificial insemination, ovum pick -up [21], IVF [22], and SCNT [9]. To date, the radioimmunoassay (RIA) system was employed to monitor progesterone concentrations in blood [23,24]. Although RIA methods provide extreme sensitivity and specificity for experimentation, the application of the method in reproduction is restricted by the radioactive half-life of reagents and the specialized equipment. In order to industrialize canine cloning, additional safety and environmentally friendly methods should be explored.

Recently, the non-radioactive electro chemiluminescence immunoassay (ECLI) was developed for antigen detection [25,26]. The method generates luminescence during electrochemical reactions in solution, and it was proven to be very useful in analytical applications as a highly sensitive and selective method. In the present study, we compared the effects of RIA and ECLI on canine ovulation judgment and the cloning of elite service dogs using ECLI as the ovulation evaluation method.

## Material and methods

### Chemicals

Unless otherwise indicated, all reagents were purchased from Sigma Chemical Co. (St. Louis, MO, USA). Chemicals used for P4 detection were ordered from Roche Diagnostics (Indianapolis, IN, USA).

### Ethics statment

In this study, 25–30-kg mixed origin, large-breed bitches, 2–5 years of age, were obtained from the canine research center of the National Institute of Animal Science (NIAS). All animal care methods and experiments were conducted in accordance with the Guide for the Care and Use of Laboratory Animals established by the Institutional Animal Care and Use Committee (IACUC) of NIAS (approval number 2015–143). Dogs were raised indoors in separate cages in a temperature-controlled room, and natural dark-light cycles were utilized. Dogs were a fed commercial diet twice daily, and were provided with water *ad libitum*. In all of the animal experiments, dogs were initially anesthetized using 6 mg/kg ketamine and xylazine, and anesthesia was maintained with 2% isoflurane. During surgery, the condition of each dog was continuously monitored using a pulse oximeter. After surgery, antibiotics (penicillin G and dihydrostreptomycin sulfate) and 2 mg/10 kg meloxicam were administered to prevent inflammation. The dogs were subsequently transferred to individual cages containing fresh water. The dogs were then monitored by the veterinarian in charge, and care was provided if the animals required specific treatments.

### Serum P4 analysis and ovulation judgment by RIA and ECLI

Blood was collected from dogs in proestrus or estrus each morning. Serum samples were separated into two parts, and P4 concentrations were detected using RIA and ECLI methods. Regarding the RIA method, serum was analyzed using a 1470 Wizard r-counter at the Seegene Medical Foundation (South Korea). A “cobas e” automatic analyzer and an ECLI kit were employed for ECLI methods, according to the user manual. Before analysis, the analyzer was calibrated using the calibrated reagents supplied in the kit. The date when serum progesterone levels reached special levels based on the experimental design was considered the day of ovulation.

### Oocyte stage judgments

After 70–72 h of hypothetical ovulation, *in vivo* cumulus oocyte complexs (COCs) obtained from oviducts were denuded and stained with 10 μg/mL Hoechst 33342, and oocytes with the 1^st^ polar body (PBI) were designated as mature. However, oocytes with the PBI and a perivitelline space >25 μm and oocytes without the PBI were designated as ageing and immature, respectively.

### Fibroblast cell culture and somatic cell nuclear transfer

Ear skin fibroblast cells were surgically collected from dogs, and were then transported to the lab within 4 h in ice-cold D-PBS. The tissue was washed three times using D-PBS, minced, and cultured in ADMEM supplemented with 10% fetal bovine serum at 38°C in a humidified atmosphere composed of 5% CO_2_ and 95% air. A fibroblast monolayer was established after being cultured for 7 d, and it was then passaged, cryopreserved in 10% DMSO, and stored in liquid nitrogen. Cells from passages 3–5 were used as donor cells for SCNT.

For nuclear transfer, canine *in vivo* matured oocytes were recovered via aseptic surgical procedures 70–76 h after the day of ovulation. Oocytes surrounded by cumulus cell layers were denuded and stained with 10 μg/mL Hoechst 33342 for 5 min. The first polar body and metaphase II spindle of denuded oocytes were removed using micromanipulation with fluorescence. One donor somatic cell was injected into the perivitelline space of each enucleated oocyte, and was then fused using electric stimulation with two pulses of DC at 3 kV/cm for 15 μs. The fused couplets were activated by calcium ionophore treatment for 5 min, and were then cultured in mSOF medium supplemented with 1.9 mmol/L 6-DMAP for 4 h.

### Embryo transfer and pregnancy diagnosis

After activation, SCNT couplets were immediately surgically transferred using a 3.5-Fr Tom Cat Catheter into the ampullary portion of the oviducts of naturally synchronous recipients. Recipients were prepared by predicting the ovulation time based on serum progesterone concentrations. Pregnancy diagnoses were assessed using ultrasound detection approximately 31 days after the embryo transfer.

### Statistical analyses

Statistical analyses were performed with SPASS (19.0, IBM, USA), and statistical significance of the P4 concentrations were analyzed using the *t*-test. The significance level was established as P<0.05.

### Experiment design

#### Experiment 1. Comparison of RIA and ECLI for the determination of serum progesterone

To compare the differences in serum P4 levels determined using RIA and ECLI methods, serum samples collected from 100 dogs were analyzed. Serum samples were separated into 10 groups based on the concentration determined by the RIA method. Colocalization and averages of the values determined using the two methods were analyzed.

#### Experiment 2. Ovulation determination based on the progesterone level detected by RIA and ECLI methods

To detect the effects of RIA and ECLI methods on the determination of the oocyte stage, 90 dogs (30 dogs for each group) were collected as oocyte donors. Serum P4 levels reached 4.0–8.0 ng/mL, which was selected as the ovulation level when RIA and ECLI methods were used. Then, a new 6.0–15.0 ng/mL level was set for ovulation determination based on ECLI methods, and oocytes stages were determined at this level.

#### Experiment 3. Dog cloning using in vivo matured oocytes and spontaneous estrus recipients determined using RIA and ECLI methods

For confirmation of whether ECLI methods could be used for dog cloning, the ovulation of oocyte donors and the estrus determination of recipient dogs were determined using RIA and ECLI, and SCNT and embryo transfer were subsequently conducted.

## Results

### Differences in progesterone concentrations determined by RIA and ECLI

**Fig 1:**
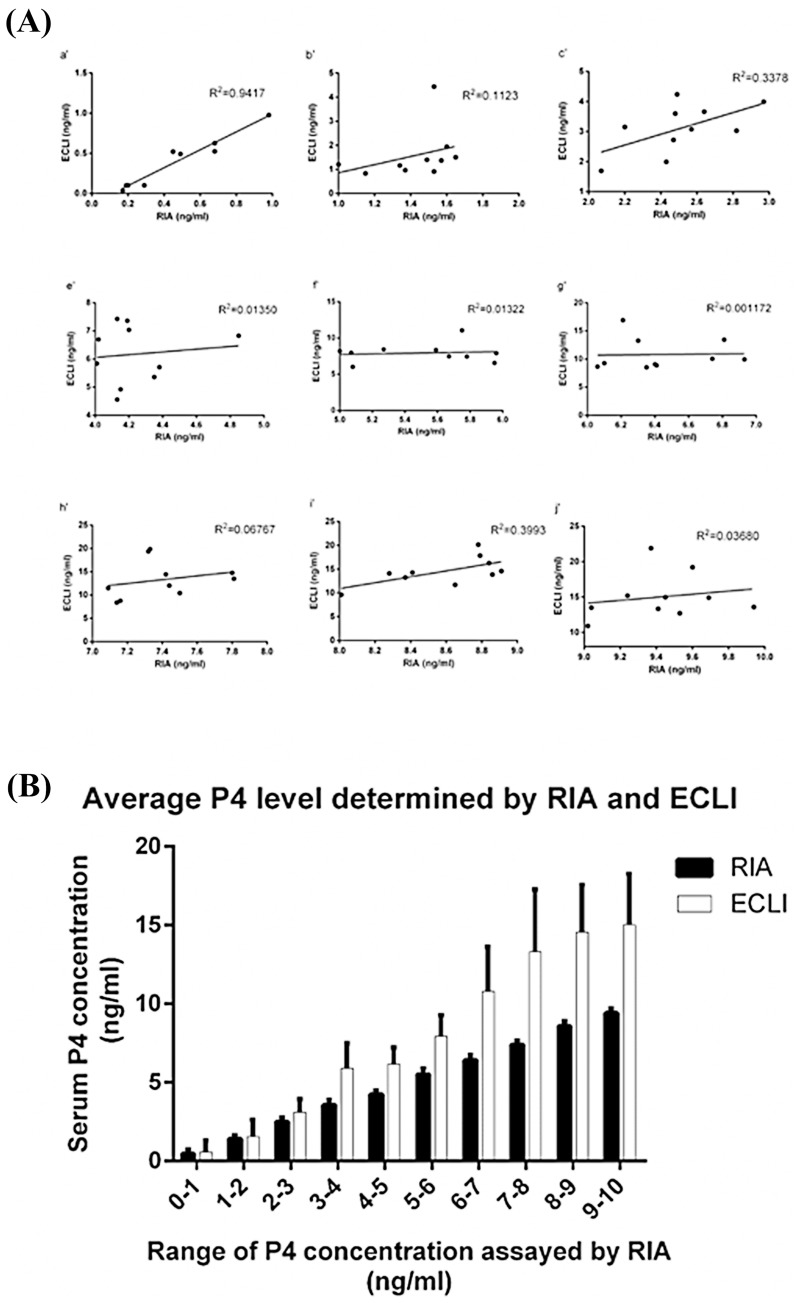
Comparison of progesterone concentrations (ng/mL) based on RIA and ECLI methods. (a'–j') Samples were separated based on the RIA data. Colocalization of RIA and ECLI methods were evaluated using the Pearson correlation coefficient. B) Average of RIA and ECLI methods. Data are presented as mean ± SD. The asterisk denotes significant differences (P<0.05).

The P4 concentrations of 88 canine serum samples, determined using RIA and ECLI, were assessed. All data were separated into different groups based on the RIA results. The results indicated that RIA and ECLI methods were consistent (R^2^ = 0.9417) at low P4 concentrations (<1 ng/mL), but they sharply decreased at high concentrations (>4 ng/mL; Fig 1A). Concentrations of P4 detected by ECLI were higher than those detected using the RIA method when the P4 concentration was higher than 3 ng/mL (Fig 1B).

### Oocyte status after ovulation judgment by RIA and ECLI methods

**Fig 2:**
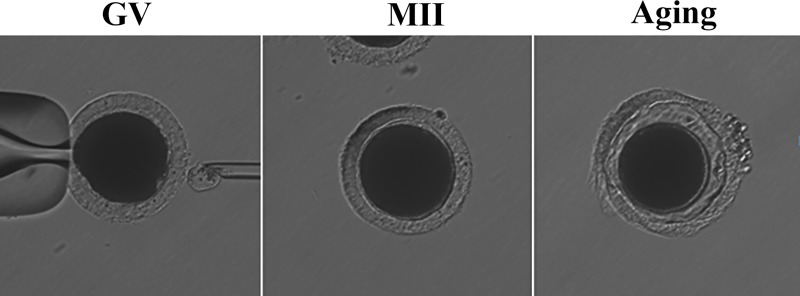
Classification of canine oocytes. Oocytes were categorized as immature, mature, and aged.

**Fig 3:**
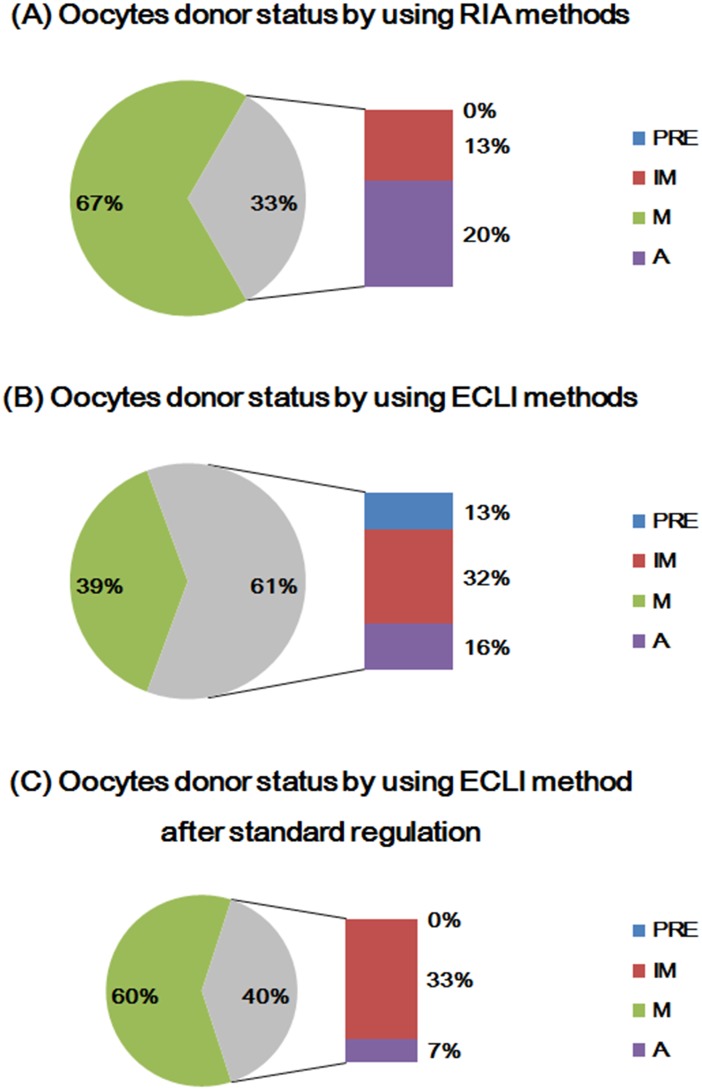
Percentage of oocyte donors at each stage detected by (A) RIA or (B) ECLI methods using P4 concentrations of 4–8 ng/mL or (C) 6–15 ng/mL as ovulation standards. Pre: preovulation, IM, immature, M, mature and A, aging. Thirty dogs were used for analysis in each group.

Immature, mature, and aging oocytes are shown in Fig 2. The P4 concentrations that reached 4–8 ng/mL were designated as ovulation. Mature oocytes were obtained from 66.67% (20 canines) and 38.71% (12 canines) using RIA and ECLI methods, respectively, and 20.00% (6 canines) and 16.13% (5 canines) of aging oocytes were obtained using RIA and ECLI methods, respectively. Only 13.33.00% (4 canines) and 32.26% (10 canines) of immature oocytes were obtained using RIA and ECLI methods, respectively (Fig 3A and 3B). The P4 level was regulated from 4–8 to a new level of 6–15 ng/mL (P4r) for ovulation prediction, because the average P4 level detected by ECLI was higher than that of the RIA method (Fig 2C). Based on the P4r levels, 60.00% (18 canines) supplied matured oocytes, and the amount of immature oocytes was reduced to 33.33% (10 canines, S1 File).

### Production of cloned canines using the ECLI system for ovulation prediction in oocyte donors and recipient canines

**Fig 4:**
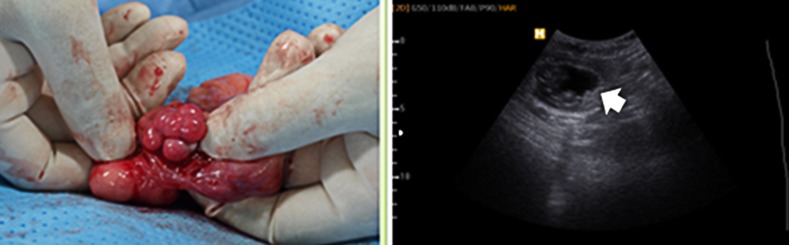
Reproductive organs of a recipient canine and an ultrasound image of a pregnant canine. Only canines at the 2^nd^ day after ovulation, as indicated by the presence of the corpus luteum, were used as recipients. After embryo transfer, pregnancy and fetal status were assessed by monitoring the fetal heartbeat. The arrows in the ultrasound image indicate gestational sac on day 3.

**Fig 5:**
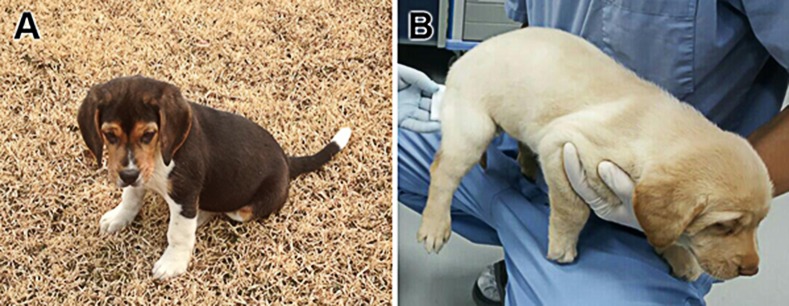
Two canines derived from SCNT. (A) Canine derived using RIA and (B) ECLI methods.

Using RIA and ECLI methods, 42 mature oocytes from eight canines and 47 matured oocytes from five canines were obtained using RIA and ECLI methods, respectively. Following SCNT, 28 and 40 fused embryos that were produced using RIA and ECLI methods were transferred to six surrogate canines. On day 31 after embryo transfer, two females were diagnosed as pregnant (pregnancy rate = 3.57% RIA and 2.50% ECLI; Fig 4), and two cloned canines (Fig 5) were delivered on Day 61 (Table 1).

**Table 1:** Canine cloned using *in vivo* matured oocytes as predicted by RIA and ECLI methods.

Method	Recipient	Recipient P_4_ conc. (ng/ml)	No. of SCNT embryos	Oocyte donor	Doner P_4_ conc. (ng/ml)	No. of pregnancies	No. of cloned pupps	Body weight
RIA	B186	6.92	10	A231, B181	11.3, 6.54	1	0	-
RIA	A162	4.46	10	A158, B147, B151	6.46, 6.68, 6.80	0	0	-
RIA	B65	5.27	8	B63, A81, B68	5.47, 5.26, 7.96	1	1	424g
ECLI	B501	9.87	12	A861	18.33	0	0	-
ECLI	A761	12.54	14	A742, A748	13.76, 10.61	0	0	-
ECLI	B459	13.23	14	B460, B468	13.12, 13.60	1	1	533g

## Discussion

In the present study, we compared the use of RIA and ECLI methods for the detection of canine serum P4 concentrations and canine ovulation judgment. The results revealed a higher accuracy with regard to P4 detection when RIA methods were employed. Although the ECLI method showed less accuracy than the RIA method, matured oocytes and a cloned canine were successfully obtained after nuclear transfer.

Canines differ from other mammals in that there is no oocyte maturation system when under *in vitro* conditions. Therefore, *in vivo* matured oocytes were collected for SCNT. After maturation, the developmental ability of oocytes underwent time-dependent degradation, which is referred to as oocyte aging [12]. Regarding the young oocytes obtained for SCNT, accurate methods for the determination of oocyte maturation *in vivo* should be developed.

Serum hormone assays are popular methods for the evaluation of mammalian ovulation. RIA is a very sensitive *in vitro* assay used to measure the concentration of hormones using antibodies. However, the radioactivity associated with the method is dangerous, and this has resulted in the limited use of this method in reproductive studies.

Enzyme immunoassays use enzyme-labeled antibodies or antigens for detection, and the method is well known in the bioanalytical field. Research conducted by Woodhead (1985) using chemiluminescence immunoassays (CLIA) has been broadly applied to clinical diagnoses and environmental analyses [27]. In the initial stages, CLIA generally used chemiluminescent indicators such as luminol, isoluminol, and acridinium ester [28,29] to directly label antigens or antibodies. Although CLIA has improved the analytical sensitivity of immunoassays, the direct labeling of chemiluminescent indicators was limited by a relatively short duration of light output. To solve this problem, ECLI was developed based on antigen-antibody conjugates using a chemiluminescent substrate, and a luminometer was used for measurements [30]. Thus, ECLI with an improved light duration output was quickly developed in recent years.

In the present study, P4 data obtained by ECLI methods displayed a higher and broader range than that obtained using RIA methods. These results could explain the high number of immature oocytes and the low number of mature oocytes recovered by ECLI methods. Prior to experimentation, all machines were calibrated using commercial calibration solutions. However, according to the manual of the analyzer, only one commercial calibration solution was employed for quality control for ECLI analyses. Therefore, the difference between RIA and ECLI might have resulted from different calibration methods. To obtain further accurate data by using ECLI in future studies, different P4 standard solutions should be employed to determine a standard curve. Based on the data from the present study, manual calibration techniques should be introduced into animal production.

Although data obtained using the ECLI method was higher than that acquired using RIA methods, more than 53% of the oocytes reached the MII stage. SCNT was performed to evaluate the feasibility of ECLI in canine cloning, and the production of a cloned canine was successful. Therefore, the results of this study indicated that ECLI methods are suitable for canine cloning.

According to our data, the P4 concentration used for ovulation judgment should be increased 1–2 units if ECLI methods are used for canine reproduction. In conclusion, ECLI is an environmentally friendly and relative accurate method for canine cloning.

## Supporting information

S1 File RAW data of oocytes donor. Percentage of oocyte donors at each stage detected by RIA (4-8ng/mL) or ECLI methods using P4 concentration of 4–8 ng/mL or 6–15 ng/mL as ovulation standards.
(XLSX)
